# Identification of FT family genes that respond to photoperiod, temperature and genotype in relation to flowering in cassava (*Manihot esculenta*, Crantz)

**DOI:** 10.1007/s00497-018-00354-5

**Published:** 2018-12-12

**Authors:** Oluwabusayo Sarah Adeyemo, Peter T. Hyde, Tim L. Setter

**Affiliations:** Section of Soil and Crop Sciences, School of Integrative Plant Science, Cornell University, Ithaca, NY USA

**Keywords:** Flowering, FT, Photoperiod, Temperature, Phylogeny, Cassava

## Abstract

Cassava is a starch-storing root crop that is an important source of dietary energy in tropical regions of the world. Genetic improvement of cassava by breeding is hindered by late flowering and sparse flower production in lines that are needed as parents. To advance understanding of regulatory mechanisms in cassava, this work sought to identify and characterize homologs of the *FLOWERING LOCUS T* (*FT*) gene. Ten members of the phosphatidylethanolamine-binding protein gene family, to which FT belongs, were obtained from the cassava genome database. Phylogenetic and sequence analysis of these proteins was used to identify two putative FT homologs which had amino acid sequences at key positions in accordance with those predicted for functional FTs. Expression of these ten genes was determined in mature leaves, immature leaves, flower buds, fibrous roots, storage roots and stem. The FT transcripts were expressed in mature leaves, as expected for their possible role in leaf-to-apical meristem signaling. In growth chamber studies, plants flowered earlier in long-day photoperiod than in short-day photoperiod. Expression studies indicated that while MeFT1 was expressed in leaves without a clear-cut photoperiod response, MeFT2 was expressed in a photoperiod-dependent manner, consistent with its involvement in photoperiodic control of flowering. In growth chambers that subjected plants to a range of temperatures from 22 to 34 °C, flowering was delayed by warmer temperatures although MeFT1 and MeFT2 expression declined in only one genotype, indicating other factors regulate this response. The earliest flowering genotype, IBA980002, had high levels of MeFT1 and MeFT2 expression, suggesting that both homologs contribute to earliness of this genotype.

**Electronic supplementary material:**

The online version of this article (10.1007/s00497-018-00354-5) contains supplementary material, which is available to authorized users.

## Introduction

Cassava (*Manihot esculenta*, Crantz) is a crop with an edible high-starch root, which grows in tropical and subtropical regions of the world. It is one of the most important staple-food crops in tropical regions and is the world’s fifth most important source of human dietary energy (FAO 2009). Flowering in cassava has received relatively little attention because in crop production systems it is propagated via stem cuttings, and the roots are harvested for consumption, rather than fruit or seed organs (Leihner 2002). However, cassava breeding is restricted because some genotypes with valuable agronomic characteristics flower extremely late and are non-synchronous with other genotypes (Ceballos et al. 2017). Conventional breeding methods and the recently developed breeding system involving genomic selection hold promise, but to be successful they require relatively rapid and synchronous flowering to speed up the breeding cycle (Ceballos et al. 2015; Heffner et al. 2009; Wolfe et al. 2017).

While flower induction is a vital developmental transition which has been studied in detail, only recently have the molecular regulatory factors been discovered (Zeevaart 2008). Floral induction is regulated by several environmental factors such as photoperiod and vernalization, which result in corresponding changes in the production of FT in leaves (Turck et al. 2008; Andres and Coupland 2012). The *FLOWERING LOCUS T* (*FT*) gene in Arabidopsis, and orthologs in all dicots and monocots studied to date, has been implicated to play a pivotal role in this floral transition. There is evidence that FT signaling plays a role in photoperiodic and developmental regulation in cassava and in closely related species in the Euphorbiaceae family. In Jatropha (*Jatropha curcas* L.), an FT homolog is expressed in all organs examined except young leaves and is thought to play a role in flower induction (Li et al. 2014, 2015). Overexpression of the Jatropha homolog in Arabidopsis hastened flowering (Li et al. 2014). In leafy spurge (*Euphorbia esula* L.), long photoperiods stimulate accumulation of FT transcripts in a diurnal manner consistent with flower induction (Hao et al. 2015). In cassava, overexpression of Arabidopsis FT elicits extremely early flowering, indicating that cassava has the necessary signaling components to interact with and respond to the FT gene product (Adeyemo et al. 2017). In addition, FT overexpression increases the prolificacy of flower production and extends the longevity of flower development. Components of the circadian clock upstream of FT have also been characterized in cassava (Adeyemo et al. 2011). FT is a member of the phosphatidylethanolamine-binding protein (PEBP) family. FT signaling is antagonized by homologs of the Arabidopsis *TFL1* gene, which are also members of the PEBP family (Li et al. 2017; Lifschitz et al. 2014; Wickland and Hanzawa 2015). Studies indicate that TFL1 is expressed in the apical meristem or other locations where its gene product competes with the FT protein by competitive binding with flowering regulatory proteins (Baumann et al. 2015; Serrano-Mislata et al. 2016; Taoka et al. 2013). In addition to FT and TFL1, another part of the PEBP family includes homologs of MFT, which play various roles in plant species; for example, in Jatropha an MFT homolog is preferentially expressed in the seed where it regulates dormancy (Tao et al. 2014).

To advance our understanding of the molecular and physiological basis of flowering in cassava under different environmental conditions, we investigated the expression of cassava *FT*-like genes. In this study, our objectives were to: (1) characterize the phosphatidylethanolamine-binding protein (PEBP) family of genes in cassava and identify putative FT-like homolog(s), (2) compare the expression of these homologs in various tissues and (3) elucidate the expression of these homologs in various experimentally controlled photoperiod and temperature environments in relation to their effects on cassava flowering. These studies deepen our understanding of likely key players of flowering regulation and other important roles in cassava, thereby advancing the potential for applications in breeding programs.

## Results

### Identification of cassava FT homologs and phylogenetic analysis

**Fig. 1:**
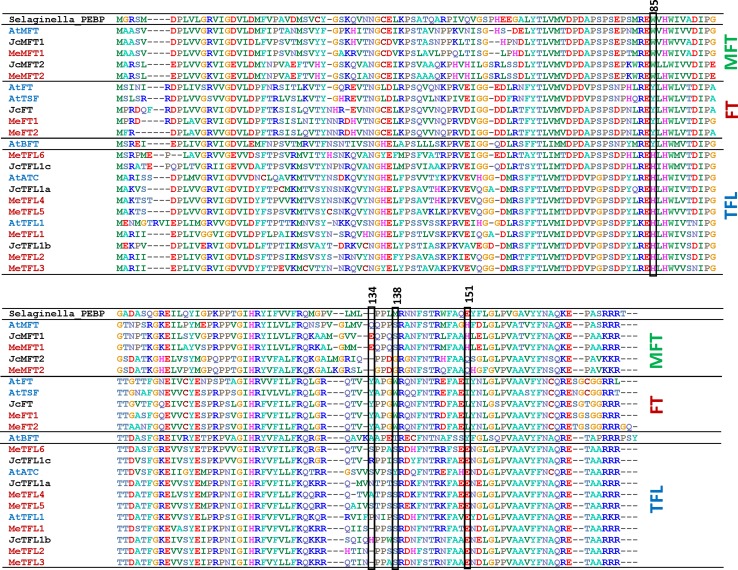
Alignment and comparison of cassava sequences with Arabidopsis and Jatropha sequences of the PEBP family. Peptide sequences are grouped into their apparent relationships to Arabidopsis MFT, FT and TFL1 proteins. Marked positions are numbered according to AtFT. Shown are sequences from *Selaginella moellendorffii* (XP_002992458.1); Arabidopsis: AtMFT (NP_173250.1), AtBFT (NP_201010.1), AtFT (NP_176726.1), AtTSF (NP_193770.1), AtTFL1 (NP_196004.1), AtATC (NP_180324.1); Jatropha (Li et al. 2014): JcFT (KF113881), JcTFL1a (KF944349), JcTFL1b (KF944350), JcTFL1c (KF944351), JcMFT1 (KF944348) and JcMFT2 (KF944352); and cassava (from phytozome.jgi.doe.gov database): MeFT1 (Manes.12G001600.1), MeFT2 (Manes.13G000800.1), MeTFL1 (Manes.09G056300.1), MeTFL2 (Manes.13G011900.1), MeTFL3 (Manes.08G024500.1), MeTFL4 (Manes.04G004700.1), MeTFL5 (Manes.11G161100.1), MeTFL6 (Manes.14G027800.1), MeMFT1 (Manes.06G008200.1) and MeMFT2 (Manes.16G019600.1)

To identify the cassava homolog(s) of Arabidopsis *FLOWERING LOCUS T* (FT), we searched the cassava genome sequence database (version 6.1, phytozome.jgi.doe.gov) using TBLASTN and the Arabidopsis FT amino acid coding sequence (AAF03936.1). Ten “hits” to the FT query with E values ranging from 2.8 e−11 to 2.7 e−35 were found. The two best matches to Arabidopsis FT (AtFT) had 77.6% and 77.8% amino acid identity to the AtFT; six were top hits when the Arabidopsis *TERMINAL FLOWER1* (TFL1) amino acid sequence was used as the BLAST query, with identities between 72 and 77%. We compared the encoded protein sequences of these ten genes with other known FT-related sequences from Arabidopsis and *Glycine max*, for which the FT/TFL gene family is well characterized, and *J*. *curcas*, which like cassava is a member of the Euphorbiaceae family (Supplementary Fig. 1). A multiple alignment of these amino acid residues revealed a high degree of conservation in the critical amino acid residues within members of this family of genes. The alignment of the cassava sequences with those in Arabidopsis and Jatropha is shown in Fig. 1. The cassava members have the conserved amino acids in positions 88 and 154 (numbered according to the Arabidopsis FT) which distinguish them as FT, TFL1, and *MOTHER OF FT AND TFL1* (MFT) subfamily members (Wang et al. 2015).

**Fig. 2:**
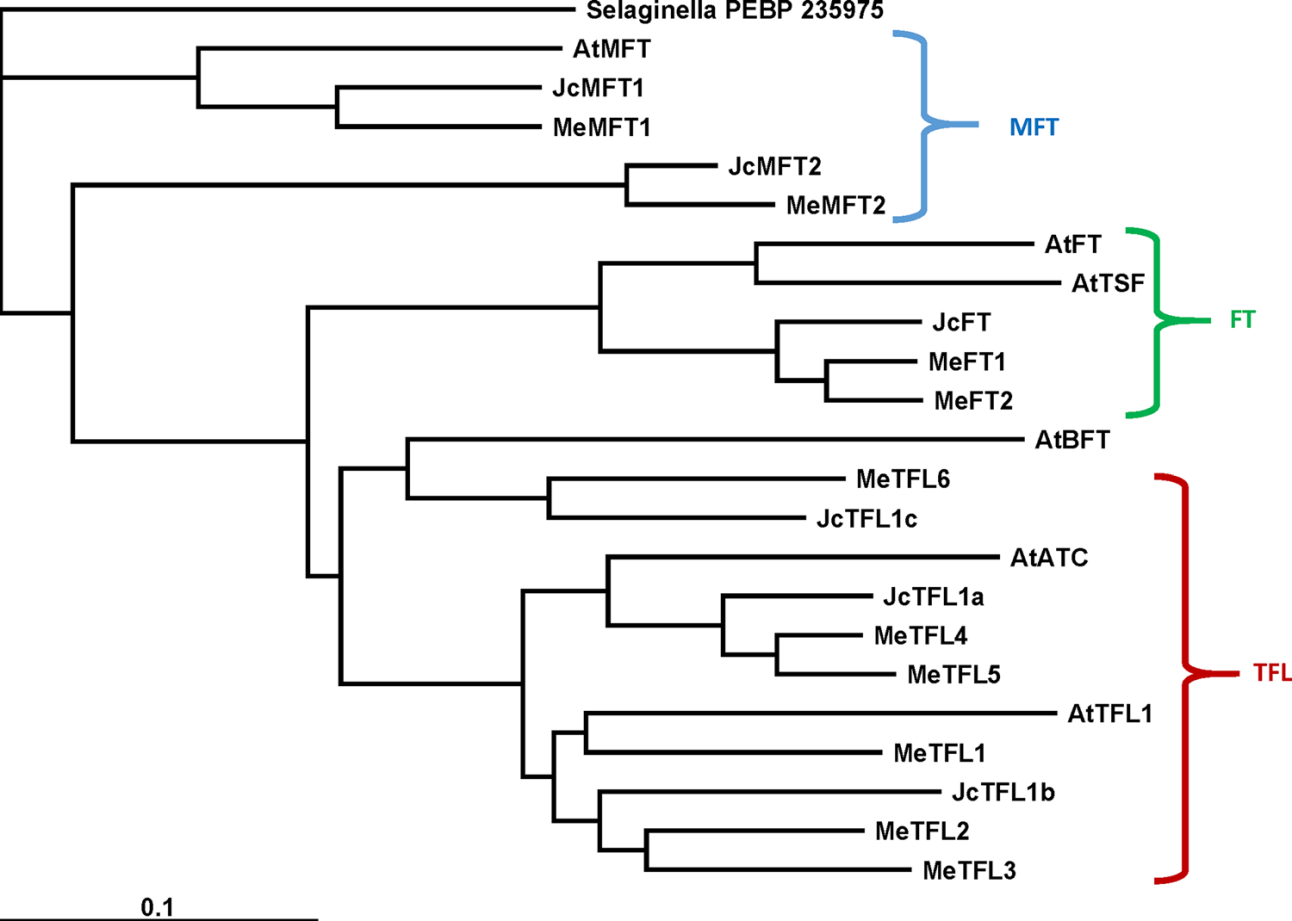
Phylogenetic tree of FT-like and PEBP-related peptides in cassava (MeFT1, MeFT2, MeTFL1, MeTFL2, MeTFL3, MeTFL4, MeTFL5, MeTFL6, MeMFT1, MeMFT2), Arabidopsis (AtMFT, AtBFT, AtFT, AtTSF, AtTFL1, AtATC) and Jatropha (JcFT, JcTFL1a, JcTFL1b, JcTFL1c, JcMFT1 and JcMFT2). Sequences were aligned with ClustalW, and the results are displayed graphically using TreeView

Amino acid alignments were used to create a phylogenic tree by the neighbor-joining method in ClustalW (Fig. 2). Among the three major subfamilies, there were two cassava sequences in the FT clade (MeFT1, MeFT2), six in the TFL clade (MeTFL1, MeTFL2, MeTFL3 MeTFL4, MeTFL5 and MeTFL6) and two in the MFT clade (MeMFT1 and MeMFT2). Cassava FTs grouped closely with Jatropha FT, but slightly more distant from Arabidopsis FT and TSF. Among the two cassava MFTs, MeMFT1 was grouped closely with Arabidopsis and Jatropha sequences AtMFT and JcMFT1, respectively. The other cassava and Jatropha MFTs were intermediate between the Arabidopsis MFT and the FT and TFL clades. Among the TFLs, there appeared to be three subclades corresponding to AtTFL1 (MeTFL1, MeTFL2 and MeTFL3), AtATC (MeTFL4 and MeTFL5), and the more distant AtBFT (MeTFL6).

### Tissue-specific expression patterns of cassava PEBP genes

**Fig. 3:**
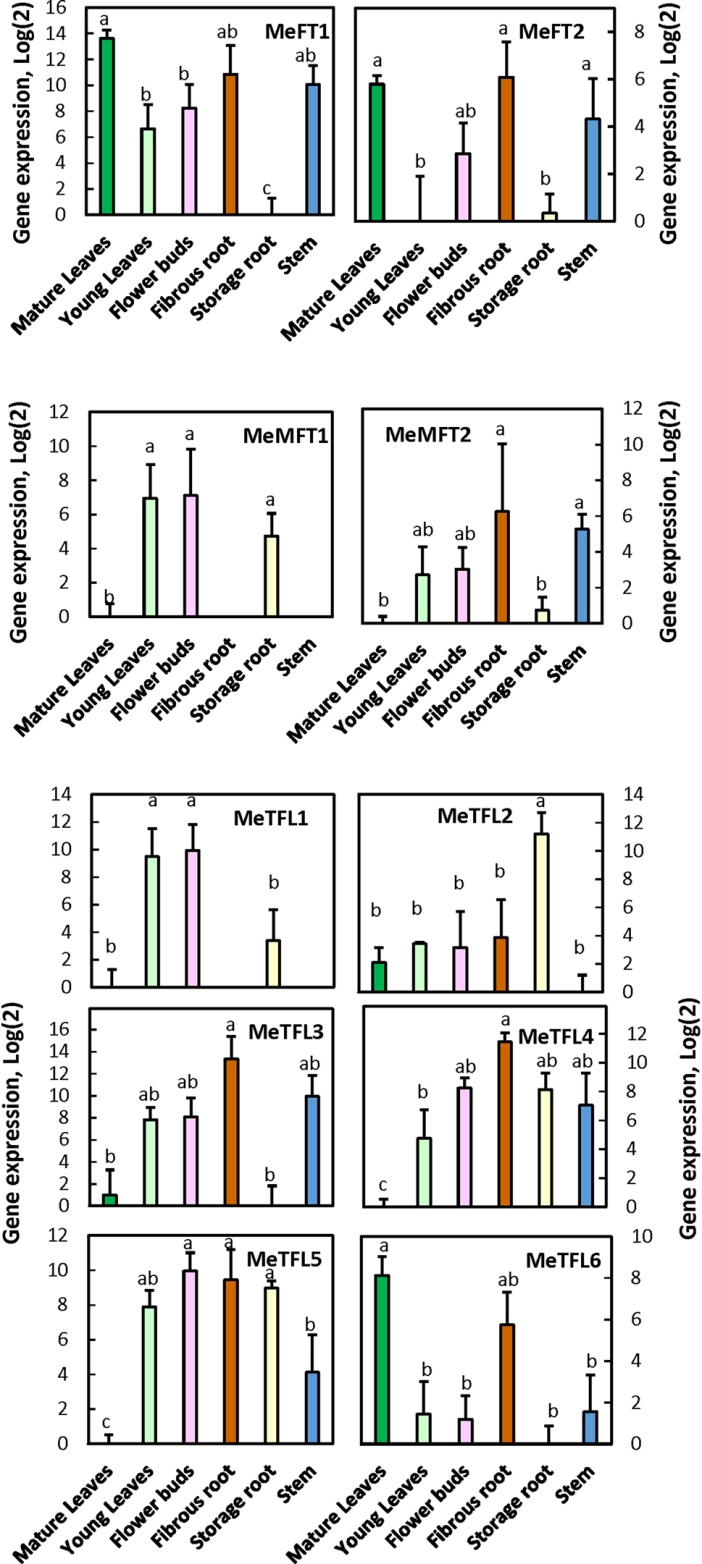
Expression of cassava FT-like transcripts (top panel), MFT-like transcripts (middle panel) and TFL1-like transcripts (bottom panel) in various organs of cassava genotype IBA980002. Transcripts were analyzed by quantitative RT-PCR using extracts from mature leaves, unexpanded young leaves, flower buds, fibrous roots, storage roots and stems. Further details are given in the Materials and methods section. Plants were grown in LD photoperiod at 28/25 °C day/night temperature. Shown are the average and SEM of 4 biological replicates of log_2_ (relative expression) for each transcript. Letters above error bars indicate significant differences (*P* ≤ 0.05) when letters differ

To explore the prospects for various functions of the cassava PEBP genes, we examined their transcript expression in different tissues of the early-flowering genotype IBA980002 using primers shown in Supplementary Table 1. MeFT1 and MeFT2 were highly expressed in mature leaves, the presumed tissue that generates the flower induction signal (Fig. 3, top panels). MeFT1 and MeFT2 were also expressed in most of the other organs, except the storage root. MeFT2 expression was absent in young leaves. In contrast to MeFT1 and MeFT2, there was negligible expression of both MeMFT1 and MeMFT2 in mature leaves (Fig. 3, middle panels). MeMFT1 expression was high in the growing tissues of young leaves and flower buds, while MeMFT2 was high in fibrous roots and stems. Also in contrast to the MeFTs, expression of all of the MeTFLs was negligible in mature leaf tissue, except for MeTFL6 (Fig. 3, bottom panels). There were no clear-cut patterns of tissue-specific expression among the MeTFLs. All tissues had expression of at least one, and often several, of the MeTFLs.

### Response of flowering and FT-like transcripts to photoperiod

To elucidate the involvement of cassava FT-like genes in regulating flowering under varying photoperiod conditions, we tested cassava genotypes with two photoperiod treatments in growth chambers: 10-h illuminated/14-h dark, versus 14-h illuminated with 10 h of full illumination at high photon flux density followed by 4 h of dim light/10-h darkness (10-h vs. 14-h daylength). The genotypes tested represented a wide range of behaviors. One of the genotypes, IBA980002, flowered much earlier than the other genotypes such that all plants had advanced to the second tier of flowering during the period of observation, while none of the Ebwanatereka plants flowered during this period (Table 1). Flower appearance was hastened by long days in all of the genotypes that flowered. Long days increased the fraction of plants that flowered within the period of observation, and among those that flowered, it decreased the average age at flowering.

**Table 1:** Response of flowering in cassava to photoperiod treatments with the indicated daylengths

Genotype	Daylength (h)	Tier	Fraction flowered (%)	Age to flowering (days)	
IBA980002	10	2nd	0	n/a^b^	
IBA980002	14	2nd	100*	145*	
Nase 14	10	1st	0	n/a	
Nase 14	14	1st^b^	67*	172*	
Nase 3	10	1st	0	n/a	
Nase 3	14	1st	33*	216*	
TMEB419	10	1st	0	n/a	
TMEB419	14	1^st^	50*	159*	
Ebwanatereka	10	1st	0	n/a	
Ebwanatereka	14	1st	0	n/a	

Shown are the proportion of plants that flowered at the indicated branch points (tier-1 or tier-2), and, for the plants that flowered, their average age at flowering (time from propagation of stem cuttings to flowering)

*Significant (*P* ≤ 0.05) effect of daylength within paired genotypes

^a^Represents flowering at the tier most closely corresponding to the sampling date. For Batch 1, it is first tier, and for Batch 2, it is second tier

^b^Not applicable

**Fig. 4:**
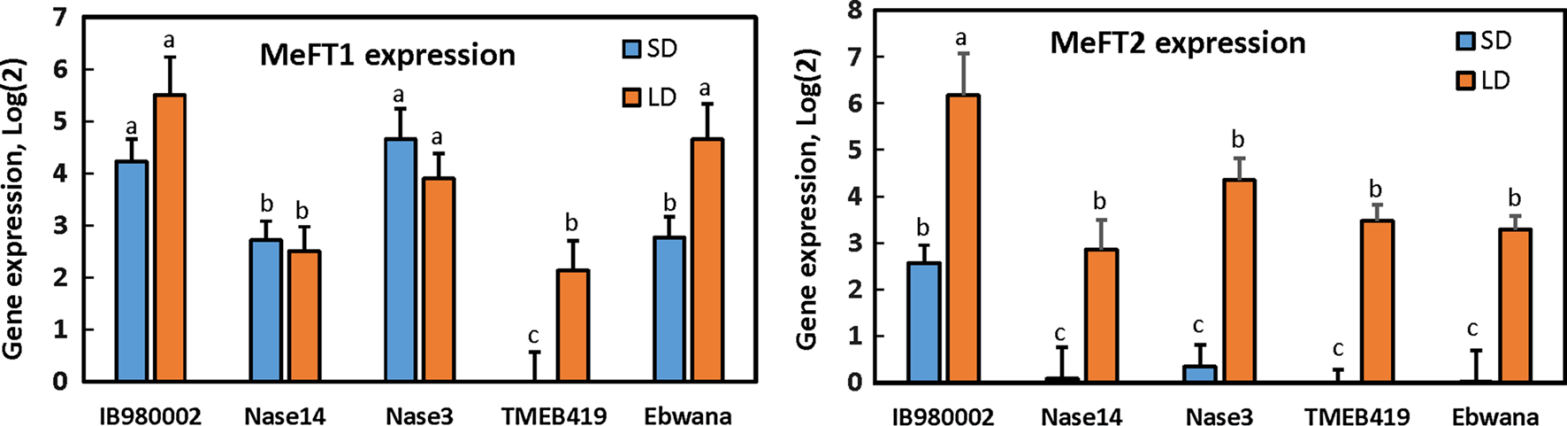
Expression of cassava MeFT1 (left panel) and MeFT2 (right panel) in response to short day (SD 10-h photoperiod) and long day (LD 14-h photoperiod). Leaf samples were sampled at the end of the light period, and transcripts were analyzed by quantitative RT-PCR. Shown are the average and SEM of 6 biological replicates of log_2_ (relative expression) for each transcript. Letters above error bars indicate significant differences (*P* ≤ 0.05) when letters differ

Expression of MeFT1 and MeFT2 transcripts was measured in mature leaves of the five genotypes under SD and LD conditions of the growth chambers. Across the full range of genotypes tested and daylengths imposed, there was no clear relationship between flower induction (Table 1) and MeFT1 expression (Fig. 4, left panel). The highest MeFT1 expression was in IBA980002, the early-flowering line, but expression was also quite high in Ebwanatereka, which failed to flower. Only TMEB419 responded to LD photoperiod with higher MeFT1 expression, corresponding to flower induction with this treatment. In contrast, for all five genotypes, MeFT2 transcript was expressed at low levels in short days whereas its expression in long days was substantially and significantly (*P* ≤ 0.05) higher (Fig. 4). Hence, MeFT2 expression was generally well correlated with the effect of long days in hastening flowering (Table 1). Even Ebwanatereka, which did not flower during the observation period, had substantially higher expression of MeFT2 in LD than in SD conditions. In the SD environment, only IBA980002, the early-flowering line, expressed MeFT2 at moderate levels, and it had the highest expression in long days.

### Response of Flowering and FT-like Transcripts to Temperature

To examine the effect of temperature on flower induction, cassava plants were grown in matched growth chambers at day temperatures of 22 °C, 28 °C and 34 °C, and night temperatures 3 °C lower in each case. The fraction of plants that flowered and the average age of plants when flowering took place were compared for two contrasting genotypes at three successive tiers of flowering (Table 2). In the early-flowering line, IBA980002, all of the plants had three tiers of flowering at 22 and 28 °C day temperatures. However, none of the plants flowered at 34 °C. First-tier flowering in the 22 and 28 °C treatments was between 75 and 82%. The Cox proportional hazard test (Cox 1972), which takes into account both the fraction of plants that flowered and the age at flowering, indicated that the 22 and 28 °C treatments were not discernibly different, whereas plants in the 34 °C treatment were significantly (*P* ≤ 0.05) different at all three tiers. In the late-flowering genotype, TMEB419, the 22 °C treatment had significantly (*P* ≤ 0.05) higher percent flowering and average age of flowering than the two warmer treatments. At 22 °C, this genotype flowered on the first tier at 145 d after planting and did not flower subsequently. There were no flowers produced in the warmer treatments at 28 and 34 °C. Hence, for both genotypes, flowering was better at cool than warm temperatures.

**Table 2:** Response of flowering in cassava genotypes to temperature treatments

Genotype	Temperature, day/night (°C)	Tier 1			Tier 2			Tier 3		
		Fraction flowered (%)	Age to flowering (days)	Cox test	Fraction flowered (%)	Age to flowering (days)	Cox test	Fraction flowered (%)	Age to flowering (days)	Cox test
IBA980002	22/19	100	75	a*	100	123	a	100	165	a
IBA980002	28/25	100	82	a	100	117	a	100	164	a
IBA980002	34/31	0	n/a	b	0	n/a	b	0	n/a	b
TMEB419	22/19	75	145	b	0	n/a	b	0	n/a	b
TMEB419	28/25	0	n/a	b	0	n/a	b	0	n/a	b
TMEB419	34/31	0	n/a	b	0	n/a	b	0	n/a	b

Shown are the proportion of plants that flowered at branch points for tier-1, tier-2 and tier-3 of fork-type branching, and for those that flowered, their average age at flowering (time from propagation of stem cuttings to flowering). Cox Proportional Hazard test results are shown for each tier

*Values within a column with different letters are significantly different (*P* ≤ 0.05) based on the Cox proportional hazard multiple comparison test

**Fig. 5:**
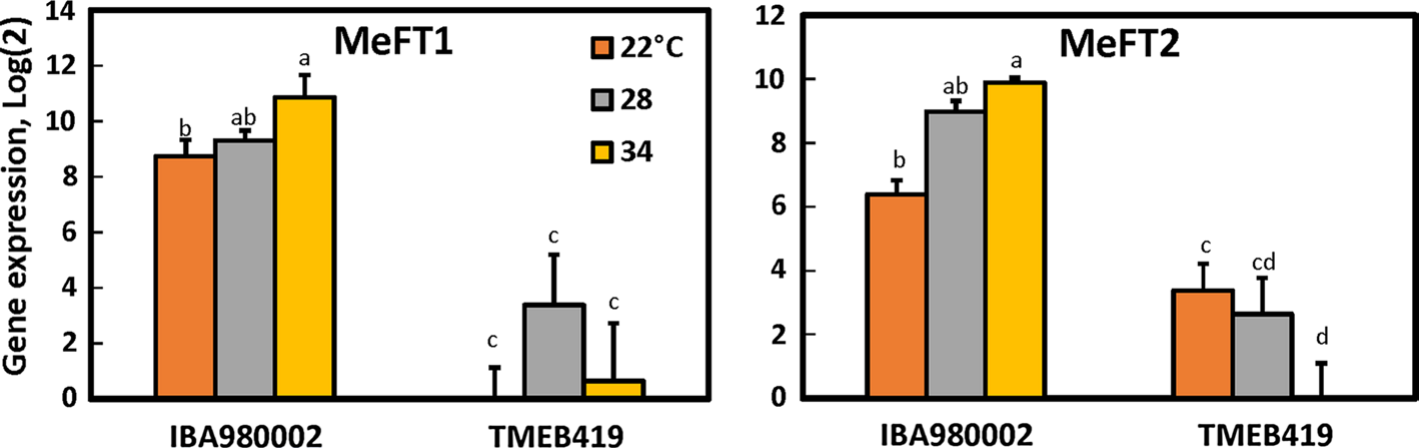
Expression of cassava MeFT1 (left panel) and MeFT2 (right panel) in two cassava genotypes (IBA980002 and TMEB419) in response to daytime growth temperatures of 22, 28 and 34 °C (night temperature 3 °C cooler in each case). Shown are the average and SEM of 4 biological replicates of log_2_ (relative expression) for each transcript. Letters above error bars indicate significant differences (*P* ≤ 0.05) when letters differ

The expression levels of both MeFT1 and MeFT2 transcripts were higher in the early-flowering line, IBA980002, than in the late-flowering line, TMEB419 (Figs. 4, 5). However, there was not a clear-cut relationship between the temperature response of flowering and the level of transcript expression (Fig. 5). In IBA980002, the trend was increasing MeFT1 and MeFT2 expression with increased temperature, which was counter to the trend of poorer flowering at increased temperature. Only MeFT2 in TMEB419 tended to have lower expression at warm temperature, which corresponded with the lack of flowering at warm temperature.

## Discussion

### Identification of cassava FT’s

In this study, we identified ten members of the PEBP gene family in cassava and compared them to homologs in Arabidopsis and *J*. *curcas*, a closely related species (Figs. 1, 2). Our goal was to identify the likely cassava FT(s) for leaf-to-apical meristem signaling. Consistent with previous findings in most other species (Wang et al. 2015), at amino acid position 85 (numbered according to Arabidopsis FT), cassava putative FTs had a tyrosine (Y), whereas cassava TFL1s all had histidine (H) at this position, and MFTs had tryptophan (W) (Fig. 1). The His residue has been shown via functional conversion studies to repress flowering while Tyr amino acid residue activates flowering (Hanzawa et al. 2005). Another defining position, residue 151 (AtFT numbering), has also been shown to convert these proteins from flowering induction to repression (Wang et al. 2015); in cassava putative FT’s, this residue was leucine (L), while cassava putative TFL1s all had glutamate (E), in accordance with previous findings (Wang et al. 2015) (Fig. 1). Cassava FT homologs were also consistent with flower induction at two other conserved sites that distinguish flower induction from repressor function (Wickland and Hanzawa 2015). At position 134, cassava FTs contain tyrosine (Y), and at position 138, cassava FTs contain tryptophan (W), consistent with the putative flower inductive function of these proteins, whereas cassava putative TFL1s contained serine (S) at position 138, consistent with a repressor function (Wickland and Hanzawa 2015).

Among the tissues analyzed, MeFT1 and MeFT2 expression was highest in mature leaves, but lower in flowers (Fig. 3), as expected for a florigen-expressing gene (Chen et al. 2018). In Arabidopsis, FT is highly expressed in the leaves, in response to photoperiodic perception occurring in this organ. However, in surveys of PEBP-family transcripts in other species, tissue-specific patterns alone are difficult to interpret with respect to function (Wang et al. 2015). In Jatropha JcFT, expression was highest in female flowers, but low in leaves (Li et al. 2015). Neither of the cassava MFT transcripts were expressed in mature leaves, and among the six cassava TFLs, only MeTFL6 was significantly (*P* ≤ 0.05) expressed in mature leaves. The role of the Arabidopsis TFL1 in repressing flowering involves its expression in the apical meristem, as has been found several species (Esumi et al. 2010; Hou and Yang 2009; Serrano-Mislata et al. 2016; Turck et al. 2008). All of the cassava TFLs, except MeTFL6, were significantly (*P* ≤ 0.05) expressed in young flowers and flower buds, in the approximate region of the apical tissue.

### Potential roles of FTs in photoperiod and temperature response

The expression pattern of MeFT2 in response to photoperiod matched that of flowering and supported the hypothesis that it plays a role in photoperiod signal development in the leaf (Fig. 4). Tissue samples for this analysis were obtained at the end of the daily light period in each treatment. In LD plants exposed to LD conditions, functional FTs are highly expressed in leaves at this time-of-day to generate a signal that elicits flowering (Andres and Coupland 2012; Turck et al. 2008). In contrast, the genotype Ebwanatereka exhibited a substantial increase in MeFT2 expression in response to LD even though flowering was not observed. However, given that this is a late-flowering line, it is possible that a longer period of observation would have revealed flowering in response to the LD photoperiod. MeFT1 expression in most of the genotypes did not respond to photoperiod, and calls into question its possible role in flowering. Only in TMEB419 did MeFT1 expression respond to photoperiod in a pattern consistent with LD flowering response. Cassava’s two FTs, with MeFT2 expression more clearly involved in photoperiod response, might be analogous to Arabidopsis where TSF and FT are close homologs, which play overlapping roles in the promotion of flowering, with FT playing the dominant role (Jin et al. 2015). Also, in soybean, GmFT2a is strictly regulated by photoperiodic changes from SD to LD, whereas the response of GmFT5a expression is gradual and low even after transfer to LD (Nan et al. 2014). In sugar beets, BvFT2 is the functional FT homolog, with negligible expression under SD conditions and gradually increasing expression in mature leaves exposed to LD, which correlates with the initiation of flowering, while BvFT1 represses floral development and its expression is promoted by SDs and inhibited by exposure to cold temperature (Pin et al. 2010; Pin and Nilsson 2012). Indeed, there are several examples in plant species where multiple FT homologs have functionally differentiated responses to environmental stimuli, and with respect to floral induction versus repression (Wickland and Hanzawa 2015).

As in many plant species, flowering in cassava was delayed when temperature was warmed in the moderate range from 22 to 34 °C (Yan and Wallace 1996) (Table 2). Studies in many species have indicated that in the moderate range of temperatures, where vernalization and thermal stress are not involved, warmer temperatures repress flowering and increase days-to-flower (Paton 1968; Yan and Wallace 1996). Cassava is well adapted to growth in hot climates, and its shoot and storage-root yield is highest at daytime temperatures of 35–40 °C (Jarvis et al. 2012). The expression of MeFT2 decreased with increasing temperature in TMEB419 in accordance with delayed flowering; however, in IBA980002 the temperature responses of both MeFT1 and MeFT2 were counter to the observed delay in flowering at warmer temperatures. Studies in some species have demonstrated FT expression levels that correlate with temperature effects on flower induction, including studies in *Fragaria* × *ananassa* (Nakano et al. 2015b), *Narcissus tazetta* (Noy-Porat et al. 2013), chrysanthemum (Nakano et al. 2015a), citrus (Chica and Albrigo 2013; Fukuda et al. 2011). Thus, it appears that in cassava additional factors were responsible for the observed effect of temperature on flowering, at least for IBA980002. Several mechanisms of temperature effects on flowering have been identified, including temperature-sensitive Phytochrome-B interactions (Jung et al. 2016), PIF4/PIF5 interactions (Thines et al. 2014) and FLM/SVP interactions (Melzer 2017; Pose et al. 2013). While PhyB and PIF mechanisms act upstream of FT expression and would be expected to affect FT levels in leaves, FLM/SVP interactions may act downstream, such as in the apical meristem. Temperature-regulated flowering could thus involve changes in such factors without affecting FT levels in leaves.

### Genotypic differences in FT expression

Cassava genotypes differed in flowering: IBA980002 flowered considerably earlier and initiated inflorescences more frequently at successive tiers; Nase 3, Nase 14, and TMEB419 flowered later; and Ebwanatereka failed to flower during the period of observation (Tables 1, 2). Both MeFT1 and MeFT2 expressions were highest in IBA980002 compared to the others in LD and SD conditions and in at all three temperatures (Figs. 4, 5). However, differences in flowering between the other four genotypes were not associated with expression levels of the FT transcripts. This suggests that genotypic differences in other components of the regulatory system for flower development are likely responsible. Indeed, analyses of flowering time in diversity trials have identified large numbers of novel genetic loci, relative to known flowering genes (Buckler et al. 2009; Li et al. 2010).

## Conclusions

We have analyzed the PEBP family of genes in cassava and identified two putative FT homologs whose amino acid sequence at key positions is in accordance with functional FTs. These FTs were expressed in mature leaves, as expected for their possible role in leaf-to-apical meristem signaling. Expression studies indicated that while MeFT1 is expressed in leaves without a clear-cut photoperiod response, MeFT2 is expressed in a photoperiod-dependent manner, and is a strong candidate to explain photoperiodic control of signaling. The earliest flowering genotype, IBA980002, had high MeFT1 and MeFT2 expression, suggesting that both homologs contribute to earliness. The genotype-dependent temperature response of flowering time and expression of these FTs suggests that other signaling factors downstream of FT are likely involved.

## Materials and methods

### Plant materials

Cassava plantlets were obtained as tissue culture explants; genotypes IBA980002 (also known as TMS I980002) and TMEB419 were obtained from the International Institute of Tropical Agriculture (IITA), Ibadan, Nigeria; genotypes Nase 3, Nase 14 and Ebwanatereka (abbreviation: Ebwana) were obtained from the National Crops Resources Research Institute (NaCRRI), Namulonge, Uganda. These genotypes are described by Bredeson et al. (2016). Seedlings were grown to full-size plants in the greenhouse and vegetatively propagated with stem cuttings for at least three growth cycles in horticultural rooting media of peat moss/vermiculite/perlite (1:1:1, by volume) to which was added by mixing: 2.2% (w/v) dolomitic limestone, 0.1% (w/v) wetting agent (AquaGro 2000G, Aquatrols, Paulsboro, NJ 08066, USA), and 2.2% (w/v) 10-5-10 Jacks Pro Media mix plus III (J.R. Peters, Inc., Allentown, Pennsylvania, USA). To initiate experiments in growth chambers, stem segments (stakes 1.5–2.5 cm dia, 10–15 cm long) from the bottom 1 m of 6-month-old plants were cut and planted into 11-L plastic pots. After 2-week growth in the greenhouse, sprouted seedlings were moved to growth chambers as described below.

### Growth conditions and environmental treatments

Growth chambers for the photoperiod experiment were Scherer, model CEL 511-38 walk-in rooms (130 × 260 × 200 cm [ht.]) with illumination by Philips cool white (4100 K) fluorescent lamps. The photoperiod experiment had two light regimes: short day (SD) with 10 h of 400 μmol photons (400–700 nm) of photosynthetically active radiation [PAR] m^−2^ s^−1^; and long day (LD) with 10 h of 400 μmol photons (400–700 nm) m^−2^ s^−1^ in light flux density with an additional 4 h photoperiod extension at 10 μmol m^−2^ s^−1^. The trials were conducted at two temperature regimens: a daytime temperature of 30 °C and a night temperature of 25 °C (Batch 1) and at a daytime temperature of 25 °C and a night temperature of 20 °C (Batch 2).

 Growth chambers for the studies on temperature and plant organs were walk-in growth rooms (135 × 245 × 180 cm [ht.]) with ten 400 W high pressure sodium and ten 400 W metal halide lamps, providing 600 μmol photons (400–700 nm) m^−2^ s^−1^. The matched chambers were model PGW 36, manufactured by Conviron Controlled Environments, Ltd (Winnipeg, Manitoba, Canada). The day time temperatures in the chambers were 22 °C, 28 °C and 34 °C, respectively, with night temperatures 3 °C lower than the day time temperatures; the photoperiod in these chambers was 12 h illuminated and 12 h of darkness.

### Alignment, phylogenetic analysis and primer design

Amino acid sequences were aligned using the maximum-likelihood method implemented in ClustalW. A neighbor-joining tree was produced from 1000 bootstrap replicates. We designed primers for the FT homologs using Primer 3 software based on the nucleotide sequences identified via BLAST searches as described in the results. The primers used in this work are shown in Supplementary Table 1.

### Tissues sampled, RNA preparation and quantitative RT-PCR for gene expression studies

For studies of growth temperature and photoperiod, mature whole leaf blades from the third from the most recently matured leaves were sampled. For the study of various plant organs and growth stages, IBA980002 plants were grown for 168 d to the third tier of flowering with 12-h photoperiod and 28 °C day temperature, as described for the temperature experiment, and tissues were sampled as follows: Mature leaves were the whole leaf blades from the third most recently matured leaves; immature leaves were leaf blades from expanding folded leaves surrounding the shoot apex; flower buds were unopened flower buds, between 2 and 3 mm diameter not including pedicel or inflorescence stalk; fibrous roots were obtained from the exposed root ball after removing plastic pots, from which growing white roots between about 1 and 2 mm were sampled; storage roots ≥ 2 cm diameter were rinsed free of soil, and blocks of tissue were sampled from the surface extending 1 cm into the core, including the cambial region; stem samples were from green stem internodes. Tissue was sampled from growth chamber plants during the last hour of illumination and immediately placed in tea bags and immersed into liquid N_2_. Tissue was ground to powder with mortar and pestle under liquid N_2_. Total RNA was extracted using a modified CTAB protocol reported by Monger et al. (2001) and quantified by absorption at 260 nm (NanoDrop ND-1000, Wilmington, DE, USA). Two μg of the total RNA was used for cDNA synthesis. Prior to the synthesis, RNA was treated with 10 U/μL DNase I (Roche) in DNase 1 buffer and incubated at 37 °C for 30 min to remove any residual genomic DNA. cDNA synthesis was performed by qScript (Quanta) and Superscript III first strand synthesis kits (Invitrogen), following the manufacturer’s instructions. Quantitative real-time PCR was performed using PerfeCTa™ SYBR^®^ Green FastMix™ (Quanta) in a Bio-Rad CFX96™ Real-Time System, C1000™ Thermal Cycler. Primers for cassava 18S RNA and for ubiquitin were used as internal controls (Supplementary Table 1). The real-time quantitative PCR was repeated with three biological replicates, and each sample was assayed in duplicate using primers listed in Supplementary Table 1. Data for the number of PCR cycles to reach the threshold (Ct) were normalized for 18S Ct values in each specimen by subtraction (ΔCt). Values were also normalized for each specimen’s UBQ Ct value, and the 18S and UBQ normalized ΔCt values were averaged. These ΔCt values were further normalized (ΔΔCt) against the genotype/treatment with the lowest quantity of each transcript in each batch, generally TMEB419, and interpreted as normalized fold expression (log_2_) assuming a PCR efficiency of 1.0. When the CT values were plotted on this log_2_ scale, they were normally distributed and subjected to analysis of variance (ANOVA), as described below.

### Flowering traits

In cassava, flowering is associated with fork-type branching which occurs via outgrowth of axillary meristems subtending the shoot apical meristem (Perera et al. 2013). After the first fork, two to four second-tier shoots develop and each of them initiates flowers at their shoot apexes (second-tier flowers). Third and subsequent tiers of flowering develop similarly. Flowering traits were recorded weekly to determine the date of flower or inflorescence appearance.

### Statistical analysis

Gene expression data for tissues and temperature had four biological replicates; for photoperiod, there were two batches (blocks), each with three biological replicates, which were subjected to analysis of variance (ANOVA) using a model for determining effects due to genotype (G), blocks (B), and effects due to interaction of T × G. Each trait was analyzed using the linear model in R (version 3.1.1, R Foundation for Statistical Computing, http://www.r-project.org/. For flowering morphology, the fraction of plants that flowered and the days to flowering were analyzed by the Cox proportional hazard test (Cox 1972).

#### Author contribution statement

OA and TS designed the experiments; TS obtained the funding; OA, PH and TS did the greenhouse and growth chamber work and associated data collection; OA performed the transcript expression analysis; OA, PH and TS analyzed the data; OA and TS prepared the tables and figures and wrote the manuscript.

## Electronic supplementary material

Below is the link to the electronic supplementary material.

Supplementary material 1 (RTF 757 kb)

Supplementary material 2 (RTF 78 kb)

## Abbreviations

FT: *FLOWERING LOCUS T*
MFT: *MOTHER OF FT AND TFL1*
TFL1: *TERMINAL FLOWER1*
Ebwana: Ebwanatereka
SD: Short day
LD: Long day
PEBP: Phosphatidylethanolamine-binding protein
