# Supplementary material for: Identification of FT family genes that respond to photoperiod, temperature and genotype in relation to flowering in cassava (Manihot esculenta, Crantz)
Source: Plant Reprod. 2018 Dec 12;32(2):181–91. doi: 10.1007/s00497-018-00354-5 (PMC6500508; doi:10.1007/s00497-018-00354-5)
Supplement: Supplementary file 1 — Supplementary material 1 (RTF 757 kb) [file 497_2018_354_MOESM1_ESM.rtf]

Supplementary Material
Identification of FT family genes that respond to photoperiod, temperature and genotype in relation to flowering in cassava (Manihot esculenta, Crantz)
Oluwabusayo Sarah Adeyemo1, Peter Hyde1, Tim L. Setter1*
Affiliations:  1Section of Soil and Crop Sciences, School of Integrative Plant Science, Cornell University, Ithaca, NY, USA
*Correspondence:  Tim L. Setter, 1Section of Soil and Crop Sciences, School of Integrative Plant Science, Cornell University, Ithaca, NY, USA;   TLS1@cornell.edu

Supplementary Figure 1. Alignment and comparison of cassava sequences with Arabidopsis, Jatropha and soybean (Glycine max) sequences, and of the outgroup Selaginella of the PEBP family.  Shown are sequences from Selaginella moellendorffii (XP_002992458.1); Arabidopsis: AtMFT (NP_173250.1), AtBFT (NP_201010.1), AtFT (NP_176726.1), AtTSF (NP_193770.1), AtTFL1 (NP_196004.1), AtATC (NP_180324.1); Jatropha (Li et al., 2014): JcFT (KF113881), JcTFL1a (KF944349), JcTFL1b (KF944350), JcTFL1c (KF944351), JcMFT1 (KF944348), and JcMFT2 (KF944352); cassava (sequences identified with prefix Me and in red font; from phytozome.jgi.doe.gov database), and soybean (sequences identified with prefix Gm, Wang et al., 2015).  Amino acid positions are indicated with reference to AtTFL1.

                                  10        20        30        40        50        60        70        80        90       100                  
                         ....|....|....|....|....|....|....|....|....|....|....|....|....|....|....|....|....|....|....|....|
Selaginella_PEBP_235975  MGRSM----DPLVLGRVIGDVLDMFVPAVDMSVCY-GSKQVNNGCELKPSATQARPIVQVGSPHEEGALYTLVMVDPDAPSPSEPSMREWVHWIVADIPG 
AtMFT                    MAASV----DPLVVGRVIGDVLDMFIPTANMSVYF-GPKHITNGCEIKPSTAVNPPKVNISG-HS-DELYTLVMTDPDAPSPSEPNMREWVHWIVVDIPG 
JcMFT1                   MAASV----DPLVVGRVIGDVIDLFVPSVTMSVYY-GSKHVTNGCDVKPSTASNPPKLTISG--HPNDLYTLVMTDPDAPSPSEPSMREWVHWVVADIPG 
Me06G008200.1 MFT1       MAASV----DPLVVGRVIGDVIDMFIPSVNMSVYY-GAKRVTNGCDVKPSLAVLPPQLTISG--LPHDLYTLVMTDPDAPSPSEPTMREWVHWIVADIPG 
Gm05g34030_MFT-like_2    MAASV----DPLVVGRVIGDVVDMFIPSVNMSVYF-GSKHVTNGCDIKPSIAISPPKLTLTG--NMDNLYTLVMTDPDAPSPSEPSMREWIHWILVDIPG 
JcMFT2                   MARSL----EPLVVGKVIGEVLDMYNPVAEFTVHY-GSKQVANGCEIKPSVAAQKPHVHILGSRLSSDLYTLVMVDPDAPSPSEPKWREWLLWIVVDIPE 
Me16G019600.1 MFT2       MARSL----EPLVVGKVIGEVLDMYNPVAEFTVHY-GSKQIANGCEIKPSAAAQKPHVHILGSRLSSDLYTLVMVDPDAPSPSEPKWREWLHWIVVDIPE 
Gm16g04830_FT-like_1     MAREN-----PLVIGGVIGDVLNPFTSSVSLTVSI-NNRAISNGLELRPSQVVNRPRVTVGG-EDLRTFYTLVMVDADAPSPSNPVLREYLHWMVTDIPA 
Gm19g28400_FT-like_1     MAREN-----PLVIGGVIGDVLNPFTISVSFTISI-NNRAISNGLELRPSQVVNRPRVTVGG-EDLRTFYTLVMVDADAPSPSNPVLREYLHWMVTDIPA 
Gm19g28390_FT-like_1     MPGGS---RNPLVVGRVIGEVIDPFEISIPFRVTY-GNREVGNGCELKPSQVANQPRVSVGG-DDLRNFYTMVLVDPDAPSPSNPNFREYLHWLVTDIPE 
Gm16g26660_FT-like_1     MPRGS---RDPLVVGRVIGDVLDPFECSIPMRVTY-NNKDVSNGCEFKPSQVVNQPRINIGG-DDFRNFYTLIAVDPDAPSPSDPNFREYLHWLVTDIPA 
Gm16g26690_FT-like_1     MPRGS---RDPLVVGRVIGDVLDPFECSIPMRVTY-NNKDVSNGCEFKPSQVVNQPRINIGG-DDFRNFYTLIAVDPDAPSPSDPNFREYLHWLVTDIPA 
AtFT                     MSINI---RDPLIVSRVVGDVLDPFNRSITLKVTY-GQREVTNGLDLRPSQVQNKPRVEIGG-EDLRNFYTLVMVDPDVPSPSNPHLREYLHWLVTDIPA 
AtTSF                    MSLSR---RDPLVVGSVVGDVLDPFTRLVSLKVTY-GHREVTNGLDLRPSQVLNKPIVEIGG-DDFRNFYTLVMVDPDVPSPSNPHQREYLHWLVTDIPA 
JcFT                     MPRDQF--RDPLVVGRVIGDVLDPFTKSISLQVTYNHR-EVNNGCELKPSQVVNQPRVDIGG-DDLRTFYTLVMVDPDAPSPSDPNLREYLHWLVTDIPA 
Me12G001600.1. FT1       MPRD----RDPLAVGRVIGDVLDPFTRSISLNITYNNRDHVTNGCELKPSQVVNQPRVDVGG-DDLRTFYTLVMVDPDAPSPSDPNLREYLHWLVTDIPG 
Me13G000800.1. FT2       MFR------DPLAVGRVVGDVLDPFTRSISLQVTYNNRDHVNNGCELKPSQVVNQPRVDIGG-DDLRTFYTLVMVDPDAPSPSDPNLREYLHWLVTDIPA 
Gm08g47820_FT-like_3     MAITT----NPLVVGRVIGDVLEPFASSIPLRVVYNNNKEVINSGELKPSQIINPPRVEVGG-DDLRTLYTLVMVDPDAPSPSDPNMREYLHWLVTNIPA 
Gm08g47810_FT-like_2     M--------DPLVLGRIIGDILDPFTSSVSLRVVYNNQSSVINSCEFKPSQIVNKPRINIRG-NDLGIFYTLIMVNPDAPSPSDPHMKEYLHWLVTNIPA 
Gm18g53690_FT-like_2     M--------DPLVIGRVVGDVLEPFTSCVSLRILYDSCSEVINCCELKPFQIINQPRVEVGG-DDFRTFYTLVMVDPDAPSPGNPNQREYLHWLVTNIPG 
Gm18g53680_FT-like_2     MPRST----DPLVIGGVIGDVLEPFTSSVSMGIVYNNCPQVINCCELKPSKILNRPRIEIGG-DDLRTFYTLVMVDPDAPSPGNPTQREYLHWLITNIPA 
AtBFT                    MSREI----EPLIVGRVIGDVLEMFNPSVTMRVTFNSNTIVSNGHELAPSLLLSKPRVEIGG-QDLRSFFTLIMMDPDAPSPSNPYMREYLHWMVTDIPG 
Gm10g08340_TFL1-like_3   MARMST---DPLIIGRVIGDVLGSFTPTIKMTVTY-NKKQVYNGYEFFPSTITTRPRVEIGG-GDMRSFYTLIMTDPDVPGPSDPYLREHLHWMVTDIPG 
Gm13g22030_TFL1-like_3   MAKMWT---DPLFIGRVIGDVLDSFTPTIKMTVTY--KKQVYNGHEFFPSTITTRPKVEIGG-GDMRSFYTLIMTDPDVPGPSDPYLREHLHWMVTDIPG 
Gm03g35250_TFL1-like_4   MARMP---LEPLIVGRVIGEVLDSFTTSTKMIVSY-NKNQVYNGHELFPSTVNTKPKVEIEG-GDMRSFFTLIMTDPDVPGPSDPYLREHLHWIVTDIPG 
Gm19g37890_TFL1-like_4   MAKMP---LEPLIVGRVIGEVLDSFTTSTKMTVSY-NKKQVYNGHELFPSTVNTKPKVEIEG-GDMRSFFTLIMTDPDVPGPSDPYLREHLHWIVTDIPG 
AtATC CENTRORADIALIS     MARISS---DPLMVGRVIGDVVDNCLQAVKMTVTYNSDKQVYNGHELFPSVVTYKPKVEVHGG-DMRSFFTLVMTDPDVPGPSDPYLREHLHWIVTDIPG 
JcTFL1a                  MAKVS----DPLVVGRVIGDVIDYFTPCMKMTVSYNSNKQVYNGHELFPSAVTHKPKVEVQGA-DMRSFFTLVMTDPDVPGPSDPYQREHLHWIVTDIPG 
Me04G004700.1 TFL4       MAKTST---DPLVVGRVIGDVIDYFTPVVKMTVSYNSNKQVYNGHELFPSAVTHKPKVEVQGG-DMRSFFTLVMTDPDVPGPSDPYLREHLHWLVTDIPG 
Me11G161100.1 TFL5       MAKTS----DPLVVGRVIGDVIDYFSPSVKMTVSYCSNKQVYNGHELFPSAVKLKPKVEVQGG-DMRSFFTLIMTDPDVPGPSDPYLREHLHWVVTDIPG 
AtTFL1                   MENMGTRVIEPLIMGRVVGDVLDFFTPTTKMNVSY-NKKQVSNGHELFPSSVSSKPRVEIHG-GDLRSFFTLVMIDPDVPGPSDPFLKEHLHWIVTNIPG 
Me09G056300.1 TFL1       MARII----EPLIVGGVIGDVLDPFLPAIKMSVSYN-SRQVHNGHELFPSTLVSKPKVEIQGA-DLRSFFTLVMIDPDVPGPSDPYLREHLHWIVSNIPG 
JcTFL1b                  MEKPV----DPLIVERVIGDVLDLFTPTIKMSVAYT-DRKVCNGHELYPSTIASKPKVAVEGD-DMRSFFTLVMTDPDVPGPSDPYLREHLHWIVSDIPG 
Me13G011900.1 TFL2       MARII----EPLIVGRVIGDVLDSFTPSIKMSVSYS-NRNVYNGHEFYPSAVASKPKIEVQGG-DMRTFFTLVMTDPDVPGPSDPYLREHLHWIVSDIPG 
Me08G024500.1 TFL3       MARII----EPLIVGRVIGDVVDYFTPEVKMCVTYN-NRQVCNGYELYPSTAVAKPKVEVQGG-DMRSFFTLVMTDPDVPGPSDPYLREHLHWVVSNIPG 
Me14G027800.1 TFL6       MSRPME--P--LAVGRVVGEVVDSFTPSVRMVITYHSNKQVANGYEFMPSFVATRPRVEIGG-EDLRTAYTLIMTDPDAPSPSDPHLREHLHWMVTDIPG 
JcTFL1c                  MSRATE--PQPLTVGRIIGEVVDAFTPSVKMSVTYNPNRQVANGHELMPSVIAAKPRVEIGG-QDLRTSYTLIMTDPDAPSPSDPHLREHIHWMVTDIPG 
Gm09g26550_TFL1-like_4   MSRLM---EQPLVVGRVIGEVVDIFSPSVRMNVTY-STKQVANGHELMPSTIMAKPRVEIGG-DDMRTAYTLIMTDPDAPSPSDPHLREHLHWTVTDIPG 
Gm16g32080_TFL1-like_4   MSRLM----EPLVVGRVIGEVVDIFSPSVKMNVTY-STKQVANGHELMPSTIMAKPRVEIGG-DDMRTAYTLIMTDPDAPSPSDPCLREHLHWMVTDIPG

 
                                 110       120       130       140       150       160       170       180             
                         ....|....|....|....|....|....|....|....|....|....|....|....|....|....|....|....|....|...
Selaginella_PEBP_235975  GADASQGREILQYIGPKPPTGIHRYIFVVFRQMGPV--LML--PPLMRNNFSTRWFAQEYFLGLPVGAVYYNAQKE----PASRRRT- 
AtMFT                    GTNPSRGKEILPYMEPRPPVGIHRYILVLFRQNSPV-GLMV-QQPPSRANFSTRMFAGHFDLGLPVATVYFNAQKE----PASRRR-- 
JcMFT1                   GTNPTKGKEILSYVGPRPPVGIHRYILVLFRQKAAM-GVV--EQPQSRANFNTRLFAAHLELGLPVATVYFNAQKE----PAAKRR-- 
Me06G008200.1 MFT1       GTNPTKGKEILAYVSPRPPVGIHRYILVLFRQRKAL-GMM--EQPQSRANFNTRMFAAHLELGLPVATVYFNAQKE----PAARRR-- 
Gm05g34030_MFT-like_2    GTNPFRGKEIVSYVGPRPPIGIHRYIFVLFQQKGPL-GLV--EQPPTRASFNTRYFARQLDLGLPVATVYFNSQKE----PAVKRR-- 
JcMFT2                   GSDATKGHELVSYMGPQPPTGIHRYVFALFKQKGALMGRIQ--PPDGRGNFNTRHFAAQSGLGLPVAAVYFNSQKE----PAVKKR-- 
Me16G019600.1 MFT2       GSDATKGHELVPYMGPQPPTGIHRYVFALFKQKGALKGRSL--GPDGRGNFSTRQFAAQHGFGVPVAAVYFNSQKE----PAVKKR-- 
Gm16g04830_FT-like_1     TTNASFGREVVFYESPNPSVGIHRIVFVLFQQLGR---DTV-ITPEWRHNFNSRNFAEINNLA-PVAAAYANCQRE--RGCGGRRY-- 
Gm19g28400_FT-like_1     TTNASFGREVVFYESPNPSAGIHRLVFILFQQLGR---DTV-ITPEWRHNFNSRNFAEINNLA-PVAAAYANCQRE--RGCGGRRY-- 
Gm19g28390_FT-like_1     TTGPNFGNEVVSYESPRPTMGIHRLVFVLFRQQFR---QRV-YAPGWRQNFNTREFAELYNLGLPVAAVFFNCQRE--SGSGGRTF-- 
Gm16g26660_FT-like_1     TTGPTFGHEVVTYENPRPMMGIHRIVFVLFRQQGR---ETV-YAPGWRQNFITREFAELYNLGLPVAAVYFNIQRE--SGCGGRRLC- 
Gm16g26690_FT-like_1     TTGPTFGHEVVTYENPRPMMGIHRIVFVLFRQQGR---ETV-YAPGWRQNFITREFAELYNLGLPVAAVYFNIQRE--SGCGGRRLC- 
AtFT                     TTGTTFGNEIVCYENPSPTAGIHRVVFILFRQLGR---QTV-YAPGWRQNFNTREFAEIYNLGLPVAAVFYNCQRE--SGCGGRRL-- 
AtTSF                    TTGNAFGNEVVCYESPRPPSGIHRIVLVLFRQLGR---QTV-YAPGWRQQFNTREFAEIYNLGLPVAASYFNCQRE--NGCGGRRT-- 
JcFT                     TTGVTFGQEIVCYESPRPSLGIHRFVFILFRQLGR---QTV-YPPGWRQNFNTRDFAELYNLGSPVAAVYFNCQRE--SGTGGRRR-- 
Me12G001600.1. FT1       TTGASFGQEVVCYESPRPSVGIHRFVFILFRQLGR---QTV-YAPGWRQNFNTRDFAELYNLGLPVAAVYFNCQRE--SGSGGRRR-- 
Me13G000800.1. FT2       TTAANFGQEVVCYESPRPSVGIHRFVFILFRQLGR---QTV-YAPGWRQNFNTRDFAELYNLGLPVAAVYFNCQRE--TGSGGRRRGQ 
Gm08g47820_FT-like_3     TTSASFGQEVVSYESPRPTSGIHRFIFVLFRQPRR---MSI-PAPGWRQNFITRDFAEYYNLGLPVAAVYFNCQRQG--GSGGRRLML 
Gm08g47810_FT-like_2     STGATTGEEIVEYESPRPTSGIHRIAFVLFRQFDR---QIV-HAPRWRQNFNTRDFAEVYNLGSPVAAVYFNCQRE--GGWGGRRR-- 
Gm18g53690_FT-like_2     TTGANFGEEVVSYESPRPMMGIHRIIFILFRQSGR---QTI-YAPGWRQNFNTRDFSEVYNLGLPVAATYFNCKRQNNSARDGRRT-- 
Gm18g53680_FT-like_2     TTGANFGEEIVSYESPRPIVGIHRIVFVLFRQLRRL---TL-QPPGWRQNFNTRDFAEIYNLGLPVAAMYFNCKRENDQS-SGRRR-- 
AtBFT                    TTDASFGREIVRYETPKPVAGIHRYVFALFKQRGR---QAVKAAPETRECFNTNAFSSYFGLSQPVAAVYFNAQRE---TAPRRRPSY 
Gm10g08340_TFL1-like_3   TTNASFGKVLVSYEMPNPNIGIHRYVFVLLKQKRR---QCVTRPPSSRDHFNTRKFSAENDLGLPVAAVYFNAQRE----TAARRR-- 
Gm13g22030_TFL1-like_3   TTNASFGNVLVSYEMPKPNIGIHRYVFVLFKQKRR---QCVTRPPSSRDHFSTRKFSAENDLGLPVASVYFNAQRE----TAARRR-- 
Gm03g35250_TFL1-like_4   TTDATFGKELVSYEIPKPNIGIHRFVFVLFKQKRR---QCV-TPPTSRDHFNTRKFAAENDLALPVAAVYFNAQRE----TAARRR-- 
Gm19g37890_TFL1-like_4   TTDATFGKELVSYEVPKPNIGIHRFVFVLFKQKRR---QCV-TPPTSRDHFNTRKFAAENDLGLPVAAVYFNAQRE----TAARRR-- 
AtATC CENTRORADIALIS     TTDVSFGKEIIGYEMPRPNIGIHRFVYLLFKQTRR--GSVV-SVPSYRDQFNTREFAHENDLGLPVAAVFFNCQRE----TAARRR-- 
JcTFL1a                  TTDATFGKEVVSYEMPRPNIGIHRFVFILFKQKRR---Q-MVNTPTSRDKFNTRKFAEENELGLPVAAVFFNAQRE----TAARRR-- 
Me04G004700.1 TFL4       TTDATFGRELVSYEMPRPNIGIHRFVFLLFKQQRR---Q-TVATPSSRDKFNTRKFAEENGLGLPVAAVFFNAQRE----TAARRR-- 
Me11G161100.1 TFL5       TTDATFGREVVSYEMPRPNIGIHRFVFLLFKQQRR---QAIVSTPSSREKFNTRKFAEENGLGLPVAAVFFNAQRE----TAARRR-- 
AtTFL1                   TTDATFGKEVVSYELPRPSIGIHRFVFVLFRQKQR---RVIFPNIPSRDHFNTRKFAVEYDLGLPVAAVFFNAQRE----TAARKR-- 
Me09G056300.1 TFL1       TTDSTFGKEVASYEIPKPNIGIHRFVFVLFKQKRR---QIIS-PPSSRDNFNTRRFATENDLGLPVAAVFFNAQRE----TAARRR-- 
JcTFL1b                  TTDATFGKEIVSYEIPRPNIGIHRFVFVLFKQKKR---SQIQHPPWSRDNFNTRNFAAENELGLPVAAVYFNAQRE----TAARRR-- 
Me13G011900.1 TFL2       TTDATFGREVVSYEIPRPNIGIHRFVFVLFKQKRR---HTIN-PPSSRDHFSTRNFAAENDLGLPVAAVYFNAQRE----TAARRR-- 
Me08G024500.1 TFL3       TTDATFGREVVSYEIPRPNIGIHRFVYLLFRQKRR---QTIN-PPASRDNFSTRNFAAENDLGPPVAAVYFNAQRE----TAARRR-- 
Me14G027800.1 TFL6       TTDASFGREVVSYETPKPVVGIHRYVFILFKQRGR---QTV-SPPASRDHFNTRRFSEENGLGLPVAAVYFNAQRE----TAARRR-- 
JcTFL1c                  TTDVSFGKEIVSYESPKPVVGIHRYVFLLFKQRGR---QTV-RPPISRDYFNTRRFAEENGLGLPVAAVYFNAQRE----TAARRR-- 
Gm09g26550_TFL1-like_4   TTDVSFGKEIVGYESPKPVIGIHRYVFILFKQRGR---QTV-RPPSSRDHFNTRRFSEENGLGLPVAVVYFNAQRE----TAARRR-- 
Gm16g32080_TFL1-like_4   TTDVSFGKEIVGYESPKPVIGIHRYVFILFKQRGR---QTV-RPPSSRDHFNTRRFSEENGLGLPVAAVYFNAQRE----TAARRR-- 
