# Supplementary material for: Identification of FT family genes that respond to photoperiod, temperature and genotype in relation to flowering in cassava (Manihot esculenta, Crantz)
Source: Plant Reprod. 2018 Dec 12;32(2):181–91. doi: 10.1007/s00497-018-00354-5 (PMC6500508; doi:10.1007/s00497-018-00354-5)
Supplement: Supplementary file 2 — Supplementary material 2 (RTF 78 kb) [file 497_2018_354_MOESM2_ESM.rtf]

Supplementary Material
Identification of FT family genes that respond to photoperiod, temperature and genotype in relation to flowering in cassava (Manihot esculenta, Crantz)
Oluwabusayo Sarah Adeyemo1, Peter Hyde1, Tim L. Setter1*
Affiliations:  1Section of Soil and Crop Sciences, School of Integrative Plant Science, Cornell University, Ithaca, NY, USA
*Correspondence:  Tim L. Setter, 1Section of Soil and Crop Sciences, School of Integrative Plant Science, Cornell University, Ithaca, NY, USA;   TLS1@cornell.edu

Supplementary Table 1.  Primer sequences used in quantitative RT-PCR.  Forward and reverse primers shown as suffixed (F, R, respectively).
Gene primer	Sequence	 	
18SF	ATG ATA ACT CGA CGG ATC GC	
18SR	CTT GGA TGT GGT AGC CGT TT	
UBQ10F	GCA ACT TGA GGA TGG CCG AA	
UBQ10R	CTC CCC TCA AAC GCA GAA CA	
MeFT1F	ATG AAA GCC CAC GAC CAT CG	
MeFT1R	GAA GTT CTG ACG CCA CCC AG	
MeFT2F	ATG CTA TGA GAG TCC ACG GC	
MeFT2R	GTA AAC AGC AGC GAC GGG TA	
MeMFT1F	GTG AAC CCA CCA TGC GAG AG	
MeMFT1R	GCA GCC CCA ACT CCA AAT GT	
MeMFT2F	CCA CTG GTG GTT GGC AAA GT	
MeMFT2R	CAC AGC CAT TGG CGA TCT GT	
MeTFL1F	TTC CAC ACT TGT TTC CAA GCC T	
MeTFL1R	AGC TCG CCA CTT CCT TTC CA	
MeTFL2F	CGG CAG TTG CTT CGA AAC CT	
MeTFL2R	TGT TCC TGG GAT GTC GCT CA	
MeTFL3F	CCA CAG CTG TTG CCA AAC CTA	
MeTFL3R	CCC AGT GCA AAT GCT CCC TC	
MeTFL4F	CAG ACA GTG GCC ACA CCA AG	
MeTFL4R	CAA CGT CTC CTT GCA GCA GT	
MeTFL5F	GCC TGG TCC AAG TGA TCC G	
MeTFL5R	GCA GGC CTA GCC CAT TTT CT	
MeTFL6F	GTC ACC GAC ATT CCT GGC AC	
MeTFL6R	CCG GTA GGC CCA ATC CAT TC	
